# Practical Notes and Formulæ

**Published:** 1885-03-20

**Authors:** 


### PRACTICAL NOTES AND FORMULÆ.

**A Local Application For Elongated Uvula.**—M. Monin ("Union Med.") employs the following mixture:

℞ Hydrochlorate of morphine....................grs. iv

Bromide of potassium..........................ʒ j

Tincture of coca...............................ʒ ij

Glycerin........................................℥ j½.

The back of the pharynx and the soft palate are to be painted with this solution, to relieve the cough caused by elongation of the uvula.—*New York Med. Jour.*

**The Treatment of Dysentery with Iodide of Phenol.**—Rosenfeld ("Centralbl. f. d. ges. Therap."; "Jahrb. f. Kinderheilk.") recommends this drug highly. He uses the following mixture:

℞ Pure iodine....................................grs. iv
Carbolic acid..................................grs. viij
Glycerin........................................℥ j.

From one to two teaspoonfuls are given, in an enema, three or four times a day. The bloody stools cease, and the tenesmus rapidly subsides under this treatment. Of a hundred and forty-two patients who were treated in this manner during an epidemic of dysentery, only six died.—*N. Y. Med. Jour.*

**Some Good Formulæ.**—As a stimulating application to a chancroid, Prof. Gross recommends:

℞ Acid tannici....................................grs. ij

Ung. hydrarg. nit..............................ʒ j

Adipis benzoat..................................℥ j.

Sig. Apply on a piece of lint.

For the sweating of phthisis, Prof. Bartholow advises:

℞ Acid gallici....................................ʒ ss

Ext. belladonnæ................................grs. ij.

Ft. pil. x.

Sig. Two pills at bedtime.

A favorite prescription of Prof. Da Costa in marked idiopathic anæmia is:

℞ Ferri sulph......................................ʒ j
Potassii carb....................................ʒ j.
M. Ft. pil. No. xl.
Sig. One after meals for first week; increase dose in second week, etc.

If the patient is a female, suspend treatment during menstruation.—*Col. and Clin. Record.*

**Treatment of Neuralgia.**—Dr. Garretson says: "In cases of the unexplainable neuralgias no single remedy has psoved so useful as these pills. My prescription is as follows:

"℞ Ferri sulphatis exsic........................
Potassii carbonatis, aa.......................gr. ccl
Syr. acaciæ..........................................q. s.

M. Ft. pil. No. 100. Sig. Begin with three a day, and increase to six; take several hundred, if necessary.—*Phil. Med. Times.*"

**For Wakefulness.**—Dr. W. S. Searle sends us the following as a remedy for wakefulness:

℞ Fluid extract cypripedium, } aa.......................℥ j

Fluid extract scutellaria, }

Fluid extract gelsemium...................ʒ ss to ʒ j.

M. One teaspoonful in water on retiring, and one more, if necessary, during the night.

This is the best remedy I have ever used for this complaint. It has proved almost uniformly efficient, even where ten grains of opium had failed.—*Pop. Science News.*

**Lactate of Quinine.**—Vigier ("Gaz. hebdom. de med. et de chir.") recommends this preparation for use in hypodermic injections, because of its solubility and the fact that it forms a neutral solution. He prefers this combination:

℞ Lactate of quinine...........................grs. xv

Glycerin.........................................ʒ vj

Distilled water..................................ʒ xx.

About seventy-five minims of this solution should be injected three or four times a day.—*N. Y. Med. Record.*

**An Ointment for Eczema of the Eyelids.**—Galezowski ("Union Med.") proposes the following:

℞ Oil of cade........................................*m* iv

Red precipitate..................................grs. j⅔

Camphor...........................................grs. iv

Vaseline ...........................................ʒ ij½.

Apply to the eczematous patches on the nose and lids of scrofulous children.—*Ibid.*

**For Bleeding Hemorrhoids.**—

℞ Pulv. aluminis.....................................ʒ ij

Pulv. camphoræ, } aa...............................ʒ j

Pulv. opii, }

Unguenti...........................................℥ j.

M. Sig. Make ointment.—*Med. World. Louisville Medical News.*

